# Does Regular Physical Activity Mitigate the Age-Associated Decline in Pulmonary Function?

**DOI:** 10.1007/s40279-022-01652-9

**Published:** 2022-02-03

**Authors:** Johannes Burtscher, Grégoire P. Millet, Hannes Gatterer, Karin Vonbank, Martin Burtscher

**Affiliations:** 1 Institute of Sport Sciences, University of Lausanne, Lausanne, Switzerland; 2 Department of Biomedical Sciences, University of Lausanne, Lausanne, Switzerland; 3 Institute of Mountain Emergency Medicine, Eurac Research, Bolzano, Italy; 4 Department of Pulmonary Medicine, Medical University of Vienna, Vienna, Austria; 5 Department of Sport Science, University of Innsbruck, Fürstenweg 185, A-6020 Innsbruck, Austria

## Abstract

Whereas the negative effects of aging and smoking on pulmonary function are undisputed, the potential favorable effects of physical activity on the aging process of the otherwise healthy lung remain controversial. This question is of particular clinical relevance when reduced pulmonary function compromises aerobic exercise capacity (maximal oxygen consumption) and thus contributes to an increased risk of morbidity and mortality. Here, we discuss whether and when the aging-related decline in pulmonary function limits maximal oxygen consumption and whether, how, and to what extent regular physical activity can slow down this aging process and preserve pulmonary function and maximal oxygen consumption. Age-dependent effects of reduced pulmonary function (i.e., FEV_1_, the volume that has been exhaled after the first second of forced expiration) on maximal oxygen consumption have been observed in several cross-sectional and longitudinal studies. Complex interactions between aging-related cellular and molecular processes affecting the lung, and structural and functional deterioration of the cardiovascular and respiratory systems account for the concomitant decline in pulmonary function and maximal oxygen consumption. Consequently, if long-term regular physical activity mitigates some of the aging-related decline in pulmonary function (i.e., FEV_1_ decline), this could also prevent a steep fall in maximal oxygen consumption. In contrast to earlier research findings, recent large-scale longitudinal studies provide growing evidence for the beneficial effects of physical activity on FEV_1_. Although further confirmation of those effects is required, these findings provide powerful arguments to start and/or maintain regular physical activity.

## Key Points

**Table:** 

Current knowledge indicates a role for physical activity to slow down the age-related deterioration of pulmonary function and associated aerobic capacity in otherwise healthy individuals.
These findings provide a further powerful argument to start and/or maintain regular physical activity.

## Introduction

Physical activity (PA) is defined as “any bodily movement produced by skeletal muscles that requires energy expenditure” [1] and includes physical exercise that is defined as planned, structured, repeated, and goal-directed PA. It is undisputed that regular PA is associated with vast health benefits. Whether it can slow down the aging-related decline in pulmonary function, commonly measured via the forced expiratory volume in 1 s (FEV_1_), however, is still debated. FEV_1_ decline starts from the fourth decade of life (age 30–40 years) with a median rate of 43.5 and 30.5 mL per year for men and women, respectively [2]. FEV_1_ is an easy to assess variable, which has been traditionally used as a surrogate measure of maximum ventilatory capacity (maximal voluntary ventilation, MVV) that was shown to represent an important predictor of dyspnea, leg effort, and aerobic capacity, irrespective of the degree of airflow limitation [3]. A close relationship between FEV_1_ and MVV maneuvers of various durations was demonstrated; indeed, longer MVV maneuvers, i.e., 60 s, lead to usage of 100% of the breathing reserve in healthy adult individuals [4]. These findings support the observed positive association between FEV_1_ and the individual aerobic exercise capacity (maximal oxygen consumption [*V*O_2_max]) in elderly subjects of both sexes that was derived from large-scale studies [5]. As FEV_1_ seems to constitute an important limiting factor of *V*O_2_max, elucidation of the interplay between PA and lung aging is of importance. Low *V*O_2_max values are known to be closely associated with increased cardiovascular events and all-cause mortality [7]. Therefore, a low *V*O_2_max, for example due to aging-related impaired pulmonary function, is a clinically relevant problem. Supporting this notion, FEV_1_ is inversely associated with the risk of functional limitations in older adults [8], and even with dementia death in a dose–response-dependent manner [9]. Whereas the negative effects of smoking on pulmonary function are undisputed [10], the potentially preventive effects of PA on the “normally” aging pulmonary system remain controversial [6, 11, 12]. Here, we assess the scientific status quo of the inter-dependencies of PA and lung aging. We discuss (1) whether and when the aging-related decline in pulmonary function limits *V*O_2_max and (2) whether, how, and to what extent regular PA can slow down this aging process and preserve pulmonary function and *V*O_2_max.

## Does the Aging-Associated Decline in Pulmonary Function Limit *V*O_2_max?

A precise interplay between pulmonary ventilation, oxygen delivery to and oxygen extraction by the working skeletal muscles is a precondition for properly matching oxygen need and demand. It is well accepted that the contribution of the cardiovascular system, and in particular, the maximal cardiac output, represents the main determinant of *V*O_2_max when utilizing large skeletal muscle groups, for example, running or cycling [13, 14]. Thus, it is not surprising that the decline of the maximal heart rate with aging is associated with a *V*O_2_max reduction [15]. Whereas diminished oxygen delivery to working muscles, resulting from the reduction and probably also maldistribution of maximal cardiac output, has been suggested as the predominant reason for the *V*O_2_max decline until late middle age, impaired utilization of oxygen by the skeletal muscles seems to play a major role in older age [16]. The latter has been attributed to impaired muscle metabolism, at least partly related to mitochondrial dysfunction, which additionally favors the development of sarcopenia [17]. Nonetheless, in certain cases, a steeper than normal decline in pulmonary function (FEV_1_), dependent on lifestyle-related risk factors, such as body mass index or smoking [18], may accelerate the age-related reduction in *V*O_2_max [5].

Despite a high demand on the ventilatory system (minute ventilation, *V*E) during maximal exercise (for instance, an incremental exercise test on a treadmill or cycle ergometer), the individual MVV usually far exceeds *V*Emax in healthy but untrained individuals [19]. MVV and *V*Emax at peak exercise are used to calculate the breathing reserve [BR = (MVV – *V*Emax/MVV) × 100], an indicator of a potential ventilatory limitation [20]. A progressive increase in mechanical ventilatory constraint to exercise hyperpnea may be a general consequence of normal aging and is associated with a tendency for expiratory flow limitation and exercise dyspnea [21]. With increasing physical fitness, the usually pre-existing ventilatory overcapacity diminishes and the pulmonary system may become limiting for *V*O_2_max in certain individuals. Here, we refer to the fact that by the use of a metabolic cart, *V*O_2_max is determined by expired *V*E, as well as by expired carbon dioxide and oxygen fractions, which explains the close association between maximal *V*E and *V*O_2_max.

It was repeatedly demonstrated that the decline in pulmonary function likely contributes to exercise limitation in both athletic young and older individuals, who can achieve high peak work rates [22, 23]. Differences in the ventilatory response to exercise in older individuals compared with younger individuals have been suggested to primarily result from the aging-induced loss of elastic recoil of the lung, thereby negatively affecting expiratory airflow rates [23]. Exercise limitations may also result from exercise-induced arterial oxygen desaturation (a 5% reduction or more from a resting oxygen saturation of about 98% at sea level) caused by insufficient hyperventilation, a shortened pulmonary capillary transit time at a high cardiac output, and/or a rightward shift of the oxyhemoglobin dissociation curve induced by lactic acidosis and hyperthermia during intense exercise [24]. Individuals with a disproportionate aging-related decline in pulmonary function may be particularly affected by increased ventilatory requirements, for example, during intense exercise at high altitude [24, 25]. In a relatively large (*n* = 1443, 714 women) cross-sectional study [5], FEV_1_ was positively associated with the individual *V*O_2_peak for both sexes (age 69–77 years) up to a certain threshold value. This threshold was located within the normal range of FEV_1_. Significant relationships between FEV_1_ and *V*O_2_max have been confirmed in several other smaller studies [23, 26, 27]. Even in patients with heart failure, in whom cardiac function would be expected to be the only *V*O_2_max limiting factor, pulmonary function indices accounted for approximately 30% of the variance in maximum exercise capacity [28]. Age-dependent effects of reduced pulmonary function (i.e., FEV_1_) on aerobic exercise performance were demonstrated also in longitudinal observations. For example, findings from a cohort of healthy individuals revealed that the decline in *V*O_2_max was predominantly explained by the age-associated decrease in both maximal heart rate and FEV_1_ [29]. In contrast to maximal heart rate, FEV_1_ may be amenable to the benefits of PA [11]. Taken together, the decline in pulmonary function likely contributes to a *V*O_2_max limitation, at least in certain individuals.

## What are the Structural and Molecular Changes Affecting Pulmonary Function with Aging?

Aging is a complex process, involving changes of all organ systems. Although FEV_1_ constitutes a “pulmonary function parameter”, various aging-related changes contribute to the FEV_1_ decline [30–34]. For example, the prevalence of hyperkyphosis in older adults varies between 20 and 40% among both sexes and is closely associated with the FEV_1_ decline [30]. In addition to the well-known age-related structural changes concerning the spine, muscles, and ribs, specific functional impairments compromise pulmonary function at an advanced age. These include decreased respiratory muscle performance, reduced cough strength, and increasing mucociliary dysfunction due to poor airway clearance associated with ventilatory restriction [35]. Aging-related changes in the alveolar-capillary membrane and in lung mechanical properties contribute to impaired lung diffusion capacity and an increased ventilation-perfusion mismatch, especially during exercise [32, 36].

The progressive deterioration of pulmonary function and structure is furthermore associated with impaired lung immunity due to declining innate and adaptive immune functions and increased proinflammatory cytokine secretion with aging [35, 37, 38]. In addition, the aging lung loses lung stem cell regenerative capacity [39] and numerous further cell-type-specific alterations contribute to the deterioration of pulmonary function. This has recently been demonstrated in rodents, in which single-cell transcriptomic and proteomic studies suggested a deregulated epigenetic control, extracellular matrix remodeling, and changes in the relative abundance of different cell types [38].

At the molecular level, impaired mitochondrial quality control, likely in part due to an age-dependent decline in autophagy, has been shown to promote the accumulation of swollen and dysfunctional mitochondria in aging type II alveolar epithelial cells [40]. Rodent studies further revealed a reduced respirational capacity (more specifically due to impaired complex IV of the mitochondrial electron transport system) of lung mitochondria with increasing age that was also correlated with elevated levels of mitochondrial DNA damage and oxidative stress [41].

The same authors [41] reported reduced levels of the central metabolism regulator nicotinamide adenine dinucleotide (NAD +) and lowered sirtuin 1 activity in the lung and other organs. Specific age-dependent metabolic shifts, in particular in conjunction with an altered relative reliance on different energy-producing pathways (i.e., glycolysis, oxidative phosphorylation, and reliance on fatty acids), are understudied but may well characterize the aging lung [42]. Support for this notion comes from the recent observation of increased expression of components of the cholesterol biosynthesis machinery in mouse type-2 pneumocytes and lipofibroblasts with aging [38]. Taken together, progressive mitochondrial dysfunction [43], oxidative stress [44], increased inflammation, and reduced immunity [45] are all general hallmarks of the aging process at the molecular level and most likely contribute to the deterioration of the aging lung, reflected by impaired pulmonary function. Remarkably, regular PA has the potential to mitigate all of these senescence-associated adverse molecular developments, as outlined in the following section. It can thus be hypothesized that PA could, at least partially, counteract the deleterious processes that reduce the function of the aging lung.

## Does PA Slow Down the Age-Related Decline in Pulmonary Function?

We here only refer to aging of otherwise healthy individuals and do not consider patients with lung diseases, for example, chronic obstructive lung disease, whose pulmonary function may often not benefit from rehabilitation programs [46]. The aging-dependent decline in FEV_1_ has been estimated to amount to 25–30 mL every year after the age of 35–40 years and may reach a 60-mL reduction per year at an age above 70 years [37], but a recent systematic review of prospective cohort studies found an even steeper decline (43.5 and 30.5 mL per year for men and women) above the age of 30 years [2]. This effect, in addition to age-related cardiovascular and musculoskeletal changes, likely contributes to the age-related decline of *V*O_2_max [5]. Notably, in rats selectively bred for high endurance capacity, lung structure was shown to be independent of habitual PA [47].

*V*O_2_max is commonly assumed to decrease with increasing age by about 10% per decade starting at the age of 30–40 years [48–50], and is thus suspiciously paralleling lung aging. Although regular PA mitigates the absolute reduction of *V*O_2_max [51, 52], it remains controversial whether this is also true for the aging-related decrement in pulmonary function [6, 12, 53–55]. In a cross-sectional study of masters athletes, no significant relationships between age-graded performance or weekly training hours and predicted lung age were observed [53]. Nevertheless, in the same study, predicted values of FEV_1_ were 9% higher and those of lung age 15% lower in athletes compared with a sedentary reference population. Similarly, McClaran and colleagues did not report beneficial influences of regular PA on the decline of resting pulmonary function in highly fit older adults, who were followed over a 6-year observation period (age 67–73 years) [12]. Despite the high fitness levels of the subjects and the rather short observation period, these findings form the basis for the assumption that exercise training would not favorably modulate the aging process of the lung [6].

In opposition to this viewpoint, several cross-sectional studies indeed indicate a slower decline of pulmonary function in older subjects who have been performing vigorous endurance exercise training on a long-term basis [56, 57], indicating that at least parts of the beneficial effects of PA on cardiorespiratory fitness are due to a mitigated deterioration of the aging lung. These observations are supported by the reported association between pulmonary function and *V*O_2_max in older people [5].

**Fig. 1:**
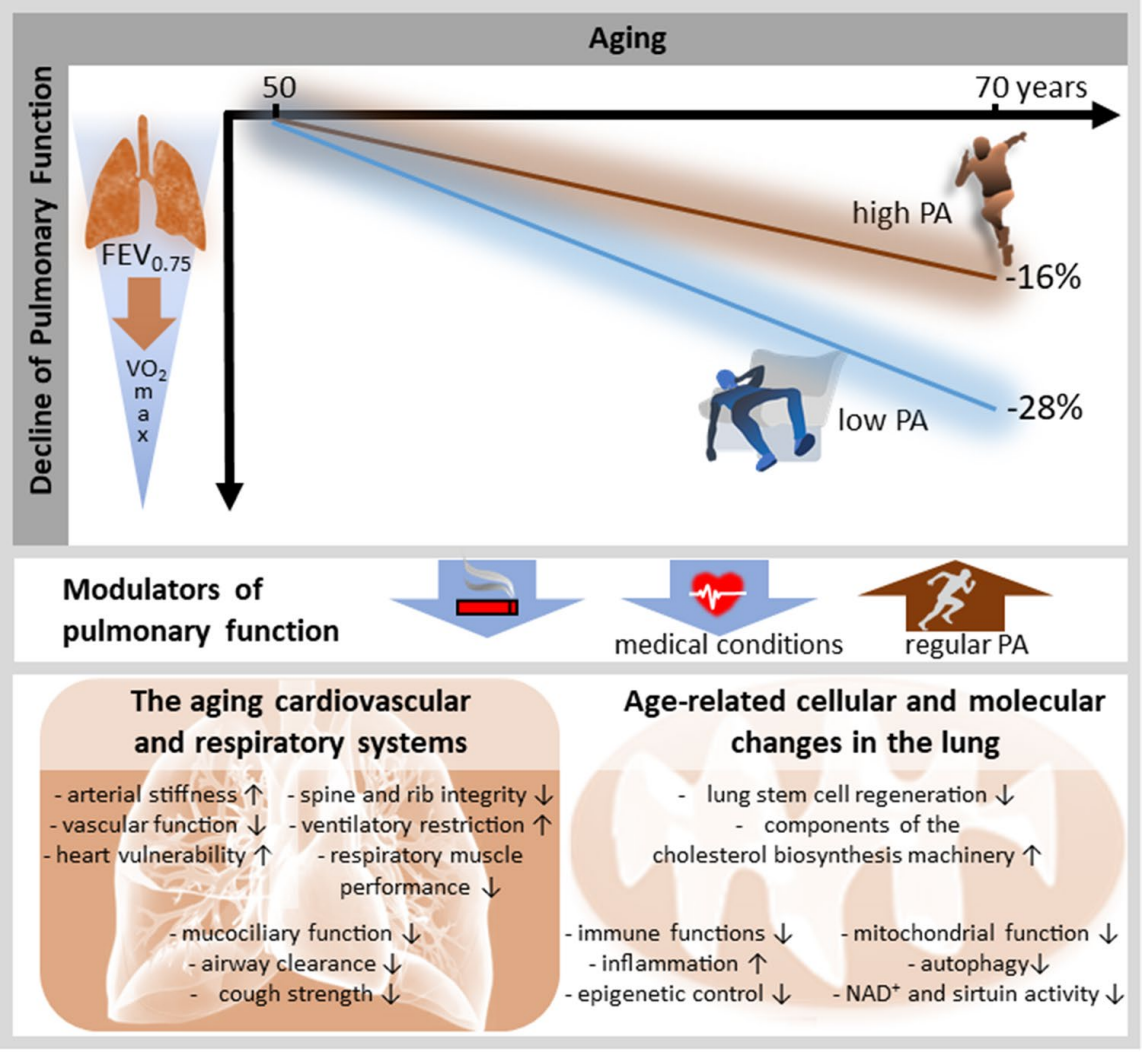
Schematic of the average percentage decline of forced expiratory volume in 0.75 s (FEV_0.75_) from 50 to 70 years of age in non-smoking individuals regularly performing high or low physical activity (PA) levels, based on reported data in Pelkonen et al. [54] (upper panel). High PA was defined as more than 1361 kJ/day and low PA as less than 734 kJ/day. The decline in pulmonary function is likely associated with a decline in *V*O_2_max [5]. Beside PA, smoking and various medical conditions are important modulators of lung aging (middle panel). Across 2 decades (age 50–70 years), continuous smokers showed a decline in FEV_0.75_ of 30% (95% confidence interval 24–37) with high PA and of 38% (95% confidence interval 32–44) with low PA [54]. As outlined in Sects. 3 and 4, complex interactions between aging-related cellular and molecular processes affecting the lung, and structural and functional deteriorations of the cardiovascular and respiratory systems may account for the concomitant decline in pulmonary function and *V*O_2_max (lower panel*)*. *NAD*^+^ nicotinamide adenine dinucleotide

Only a few longitudinal studies have evaluated the effects of PA on aging-related changes in pulmonary function, while also considering important confounding factors such as smoking habits. For instance, longitudinal results derived from a Finnish cohort and comprising individuals of different PA levels highlight the relationship between PA and a slower decline in pulmonary function (FEV_1_) in middle-aged and older people [54]. This study nicely demonstrated the longitudinal (over 20 years) effects of aging, smoking, and regular PA in men on pulmonary function, i.e., the forced expiratory volume in 0.75 s (Fig. 1).

Another prospective follow-up study found that moderate-to-high levels of regular PA were associated with reduced pulmonary function decline and the risk of chronic pulmonary disease among smokers [58]. Evidence for the beneficial effects of PA on pulmonary function also comes from recently published findings of two large-scale longitudinal studies. The “Canadian Longitudinal Study on Aging” indicates that replacing sitting time with PA leads to significant improvements in FEV_1_ among healthy adults and those with respiratory disease as well [11]. Similarly, “The English Longitudinal Study of Aging” demonstrated that individuals who remain physically active, or become active at an older age, maintained or improved their pulmonary function (forced vital capacity and FEV_1_) [55]. It seems likely that PA counteracts the stiffening tendency in the chest wall [54], as older endurance athletes have been suggested to experience less aging-related effects on lung elastic recoil and diffusion surface [59]. As regular PA was found to be positively associated with diaphragm muscle thickness, attenuation of the age-related decline in respiratory muscle strength may represent a further mechanism contributing to the preservation of a high ventilatory capacity [60]. Findings from “The English Longitudinal Study of Aging” extend the spectrum of mechanisms potentially responsible for PA-related benefits on pulmonary function by indicating that such effects may be mediated by lowering systemic inflammation (e.g., extent of C-reactive protein). Exercise also induces a fortification of antioxidant defences and the resulting enhanced capacity to manage oxidative stress is likely involved in the attenuation of age-related decline of pulmonary function as well [61].

Regular PA can mitigate many aspects of the aging process, from molecular to systemic. Physical activity has been demonstrated to preserve mitochondrial health, both in the skeletal muscle [62–64] and other organ tissues, including the lung [65]. Generally, PA bolsters the cellular anti-oxidative capacities [66–69] and it has also been shown to attenuate aging-related deficiencies of anti-oxidative defense systems [67]. As the restoration of redox homeostasis (by genetic and pharmacological inactivation of the reactive oxygen species producing NADPH oxidase-4) ameliorated aging-related lung fibrosis in mice [70], the oxidative stress-mitigating effects of exercise may explain some of the benefits regular exercise confers specifically on pulmonary function. Moreover, PA also mitigates increasing inflammation at a higher age [71]. Finally, there is some evidence that PA increases resilience to lung injury and infection. Two weeks of exercise, for example, prevented mitochondrial damage and the formation of edema in the lungs in a rodent model of experimental lung injury [65]. In summary, there is growing evidence for a beneficial role of PA on the aging process of the pulmonary system.

## How Much Does PA Slow Down the Age-Related Decline in Pulmonary Function?

A recent, large, multicenter cohort study addressed this question (in smokers) and demonstrated a linear increase of FEV_1_ with both frequency (up to four or more times per week) and duration (up to ≥ 4 h per week) of usual vigorous PA [72]. These findings support the advice to meet current public health guidelines, which recommend at least 150–300 min of PA at moderate intensity or 75–150 min at vigorous intensity per week [73]. Nevertheless, sophisticated longitudinal studies are needed to elucidate how different types of PA, including exercise training, impact on the aging process of the pulmonary system over the course of a lifetime in both sexes.

## Conclusions

A larger than normal decline in pulmonary function (i.e., FEV_1_) in otherwise healthy aging individuals may limit *V*O_2_max. In very fit people, this effect may become apparent relatively early due to their greater ventilatory requirements. It is well established that even FEV_1_ threshold values above the lower limits of normal pulmonary function modulate individual *V*O_2_max levels, and thus also morbidity and mortality from various diseases. Importantly, recent research provides growing evidence for the beneficial effects of regular PA on factors related to pulmonary function and consequently on *V*O_2_max and associated health benefits. Although well-designed studies are required to confirm this preliminary evidence, the current knowledge indicates that PA might slow down the age-related deterioration of pulmonary function and thus provides a further powerful argument to start and/or maintain regular PA.
